# Robust Prediction of Single and Multiple Point Protein Mutations Stability Changes

**DOI:** 10.3390/biom10010067

**Published:** 2019-12-31

**Authors:** Óscar Álvarez-Machancoses, Enrique J. De Andrés-Galiana, Juan Luis Fernández-Martínez, Andrzej Kloczkowski

**Affiliations:** 1 Group of Inverse Problems, Optimization and Machine Learning, Department of Mathematics, University of Oviedo, C. Federico García Lorca, 18, 33007 Oviedo, Spain; UO217123@uniovi.es (Ó.Á.-M.); andresenrique@uniovi.es (E.J.D.A.-G.); jlfm@uniovi.es (J.L.F.-M.); 2 Department of Computer Science, University of Oviedo, C. Federico García Lorca, 18, 33007 Oviedo, Spain; 3 Battelle Center for Mathematical Medicine, Nationwide Children’s Hospital, Columbus, OH 43205, USA; 4 Department of Pediatrics, The Ohio State University, Columbus, OH 43210, USA

**Keywords:** protein mutation, machine learning, holdout sampler, mutation stability, neural network

## Abstract

Accurate prediction of protein stability changes resulting from amino acid substitutions is of utmost importance in medicine to better understand which mutations are deleterious, leading to diseases, and which are neutral. Since conducting wet lab experiments to get a better understanding of protein mutations is costly and time consuming, and because of huge number of possible mutations the need of computational methods that could accurately predict effects of amino acid mutations is of greatest importance. In this research, we present a robust methodology to predict the energy changes of a proteins upon mutations. The proposed prediction scheme is based on two step algorithm that is a Holdout Random Sampler followed by a neural network model for regression. The Holdout Random Sampler is utilized to analysis the energy change, the corresponding uncertainty, and to obtain a set of admissible energy changes, expressed as a cumulative distribution function. These values are further utilized to train a simple neural network model that can predict the energy changes. Results were blindly tested (validated) against experimental energy changes, giving Pearson correlation coefficients of 0.66 for Single Point Mutations and 0.77 for Multiple Point Mutations. These results confirm the successfulness of our method, since it outperforms majority of previous studies in this field.

## 1. Introduction

The amino acid sequence of a protein is the most important factor that determines its secondary and tertiary structure, dynamics and, ultimately, its function. The understanding of the mechanisms that determine protein stability is one of the forefront challenges in proteomics and transcriptomics, since even a single amino acid substitution can be the cause of a devastating disease [1] Experiments are utilized to engineer or design proteins with specific mutations to examine the effect of that specific amino acid substitution [2]. The effect of a mutation is assessed by (ΔΔG)—a measure of the change in free energy between the folded and unfolded states when a point mutation is present. This has been found to be an excellent indicator of whether a point mutation is favorable in terms of protein stability. A comprehensive database of experimentally obtained mutations and its associated free energy changes is available at ProTherm database [3]—the thermodynamic Database for Proteins and Mutants that contains more than 10,000 data of several thermodynamic parameters for wild type and mutant proteins. Each entry in ProTherm includes data for unfolding Gibbs free energy change, enthalpy change, heat capacity change, transition temperature, activity and structural information such as secondary structure and solvent accessibility of wild type residues.

Since mutagenesis experiments are expensive and time-consuming, the amount of available experimental data is limited [4]. Therefore, computational methods have been proposed to predict stability changes observed in a mutant protein in comparison to the wild type [5,6,7,8]. Generally speaking, the five main computational approaches can be categorized as: (1) Physical potential-based methods, (2) statistical potential-based methods, (3) empirical potential-based methods, (4) combinatorial and rigidity-based methods and 5) machine learning approaches [9,10].

Physical potential-based approaches utilize Molecular Dynamics simulations of proteins using atomic force-fields, such as CHARMM, GROMOS or AMBER. Such simulations are however costly when applied to large datasets [11]. Statistical potential-based methodologies rely on the use of statistically-derived potentials [12,13,14] from Molecular Dynamics simulations [15], environmental propensities [16], substitution frequencies or correlations of residues forming non-bonded contacts exposed to solvent [17,18,19]. Empirical potential-based methods utilize a weighted combination of physical terms and structural properties for fitting experimental data [20,21,22].

Combinatorial and rigidity-based methods explore the effects of mutations by estimating the value of configuration entropy for a rigid cluster [23]. Rigidity analysis is a combinatorial technique that identifies protein regions that are flexible or rigid [24] and has the advantage of not relying on protein homology or on costly all-atom energy calculations [25,26]. Machine learning methods are more flexible and combined with rigidity analysis improve substantially predictions of protein stability change upon mutation [27].

Machine learning is a branch of artificial intelligence relying on detection of patterns and inference in data. Machine learning algorithms build a mathematical model based on sample “training data”, in order to make predictions for new data. This approach has been used in the past to infer the effects of protein mutations. Capriotti et al. [9] and Chen et al. [10] used Support Vector Machines to infer the sign of the stability change for a protein upon a single-site mutation. Dehghanpoor et al. [28] predicted the effect of single site and multiple site mutations using Support Vector Machines and Random Forest. Furthermore, data from amino acid replacements that are tolerated within members of the same protein family were used to devise stability scores and implemented in an online web server [29,30,31].

Machine learning methods have also been widely utilized in proteomics in the prediction of methylation sites [32], phosphorylation sites [33], and other post-translational modifications, and in prediction of protein subcellular locations [34]. Jia et al. [35] developed machine learning tools to obtain models of protein mutants based on thermostability data to assess impacts of mutations. The machine learning methods employed to study mutations include: Support Vector Machine, Random Forest, Naive Bayes classifier, k-Nearest Neighbor, Neural Network, and Partial Least Squares approaches, with the Random Forest Approach having the highest accuracy. Li et al. [36] developed a Random Forest algorithm in order to predict stability changes due to single and multiple amino acid substitutions. This method was further improved by Dehghanpoor et al. [28] by combining it with protein rigidity calculations to boost the prediction performance.

Recent improvements are based on a multiple classifier approach. A natural way of combining multiple classifiers is to create stacked ensembles, which utilize the output of many different machine learning models, all trained on the same task, to develop a new learning model [37,38,39].

The approach used here by us and presented below differs from previous methods due to fact that the classifiers are trained in different random bags.

In the present paper, we present an efficient and systematic machine learning and uncertainty analysis-based methodology for predicting the effect of amino acid substitutions on a protein structure. Our method is based on the fact that every amino acid substitution has its inherent Cumulative Distribution Function (CDF) of energy changes. Energy is sampled via a Random Holdout Sampler to obtain the energy distribution of the mutant protein, which indicates uncertainty of the energy of a mutant. This sampler has been used earlier in different fields to assess the intrinsic uncertainty in the inverse problem and in various classification problems [40,41,42].

The combination of the energy distribution of the mutant, the observed change of energy, and information on the specific amino acid substitution and the residue position at which the substitution takes place are utilized to train a simple Neural Network to predict the effect of a mutation on a given protein (ΔΔG) Results are, later, validated utilizing experimental data extracted from the ProTherm database.

The data set of proteins with two mutations in ProTherm database is smaller than the set of proteins with single mutation, however, it is sufficiently large to give us a good insight on the uncertainty associated with the energy change caused by a mutation.

Advantage and robustness of our methodology is achieved via the use of a large number of Holdouts and through multiple trainings and validations of the Neural Network to compute the frequency histogram of accuracies. This approach leads to better prediction accuracies than any other machine learning method used in the past. Furthermore, our method often achieves a robust performance in the prediction of the effect of single and multiple mutations while being fast enough to be run in a few minutes. Remarkably, our methodology is distinguished from others since our machine learning-based method does not require the computation of energy or other physical properties. Therefore, like in the work of Dehghanpoor et al. [28], it is possible to predict the effect of a mutation without the need of biophysical information and the method does not depend on hydrogen bond energies, van der Waals forces and generally no force field is needed. Our present study extends past work in this field by introducing the concepts of distribution of mutation energy and its uncertainty and by parametrization of a Neural Network in order to accurately predict the observed energy. Therefore, it represents is highly novel approach to prediction of changes of stability of proteins due to mutations.

## 2. Methods

### 2.1. Protein Datasets

The protein dataset has been derived from the Thermodynamic Database for Proteins and Mutants (ProTherm) which contains approximately 14,500 data of several thermodynamic parameters for wild type and mutant proteins along with detailed information on experimental methods and conditions. The thermodynamic data is linked to sequential and structural data in Protein Data Bank (PDB), Protein Information Resource (PIR) and SWISS-PROT [43].

In this paper, two different datasets are utilized. The databases utilized are randomly split for learning and testing for both the uncertainty analysis of mutant proteins and for learning and blind validation of the Neural Network. That way, the machine learning process conforms to the norms that are accepted in the AI community of independent training and generalization.

The databases have been derived from ProTherm by taking into consideration the following assumptions: (1) the change in protein free energy (ΔΔG) has been measured experimentally and deposited in the ProTherm database; (2) the protein structure is known and has been deposited in the Protein Data Bank (PDB) [44]; (3) the data is limited only to single point and double point mutations.

The Single Point Mutation Dataset is shown in Supplementary Material 1, and the Double Point Mutation Dataset in Supplementary Material 2.

### 2.2. Protein Mutation Prediction Methodology

#### 2.2.1. Single Point Mutations

In this paper, we propose a protein mutation prediction scheme composed of two steps. Our methodology is based on the assumption that every amino-acid substitution has its own Cumulative Distribution Function (CDF) of changes of energy. Therefore, regardless of the protein in which that substitution takes place, the produced energy change can take on any value provided by CDF. In other words, CDF of changes of energy provides a set of admissible changes of energy that a given mutation may cause in a specific protein.

In this sense, the first step consists of sampling the uncertainty of energy change landscape for a given amino acid substitution in order to obtain the overall change of energy distribution for the mutant protein (also considered as the uncertainty). This initial step is carried out via the Holdout Random Sampler, which has been proposed by Cernea et al. [42] in application to the problem of phenotype prediction. It is based on the boot-strapping technique. This algorithm quantifies the cumulative distribution function of the change of energy of a mutant protein on a validation dataset with the aid of the landscape of possible energy changes that a mutation causes regardless of protein and the mutant residue position in the protein. This mutation energy landscape was previously extracted from a learning dataset [40,41].

The second step consists of predicting the energy change produced by the amino acid substitution with a Neural Network composed of one hidden layer and 10 neurons. The Neural Network is trained considering CDF of changes of energy of mutant proteins extracted from the Holdout Sampler, and, later on, validated by comparing with the real energy changes extracted from ProTherm database.

**Figure 1:**
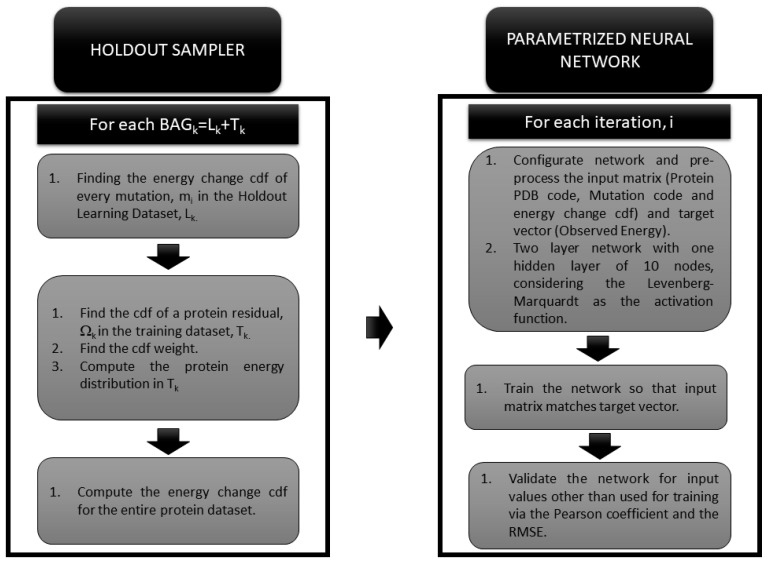
Workflow for the Prediction of Single Point Protein Mutation Energy Change and its Uncertainty.

An illustrative general workflow of the protein mutation prediction scheme is shown in Figure 1. Further details of the algorithm are shown in the following sections.

#### 2.2.2. Multiple Point Mutations

Prediction of protein stability for Multiple Point Mutations follows a similar scheme as in the prediction of stability for single point mutations. It is also based on the fact that every amino acid substitution has its own CDF of energy changes. Consequently, if the single point amino acid distributions are taken and Holdouts of multiple point mutations are generated, it is possible to quantify the cumulative distribution function of energy changes for a mutant protein using a validation dataset with the aid of the landscape of possible energy changes that a mutation causes, regardless of protein, and the position of the mutated residue in the protein.

The CDF of changes of energy consists of a set of parameters that improve the accuracy of a Neural Network, composed of one hidden layer and 10 neurons. The Neural Network is trained by considering the CDF of changes of energy upon amino acid mutation of proteins extracted from the Holdout Sampler, and, validated with the experimentally observed energy changes extracted from ProTherm database.

### 2.3. The Holdout Sampler-Based Uncertainty Predictor

The purpose of our algorithm is to explore the change of energy landscape for every mutation. The sampling of the energy landscape is, later used to compute the energy landscape for a specific mutant protein. The simplest way of carrying out this task is by utilizing random data bags with different datasets for training, followed by a testing procedure. This is comparable to modifying the evidence of $\;c^{\mathrm{obs}}$ with respect to the classifier/regressor $\;L^{*}$, since part of the samples used for blind testing/validation have not been used for training. L* corresponds to the classifier/regression model utilized to predict the free energy changes or free energy uncertainty, while $\;c^{\mathrm{obs}}$ is the observed free energy change, coming from experiments. The problem consists of finding the uncertainty of protein mutation free energy changes relative to $L^{*}\left(g\right)$ can be interpreted as a generalized regression problem of the observed free energy change, $c^{obs}$ with respect to the predicted CDF.

This method is based on the statistical technique of bootstrapping, or arbitrary sampling with replacement [45], which is used to build the confidence intervals in sample estimates and to estimate the sampling distribution of any statistic via a random sampler.

In prior works for other application, this methodology was utilized to optimally sample model parameters of posterior distribution via the least squares fitting of different data bags [40,41]. In this case, the approach is similar; the idea is to sample the model parameters in order to obtain effect of mutation on distribution of energy. The Holdout Sampler samples CDF of energy change of the mutant protein as follows:

Data bagging: We randomly divide the data set into a 75/25 data bag holdouts, where 75% of the data is used for learning and 25% for testing/validation. In this case, 100 different bags were generated. For each holdout, let us consider an amino acid substitution, $m_{i}$, which is present in a set of proteins, $p_{k}$. That set of protein has an associated free energy change, which is known, $E\left(p_{k}\right)$, therefore; it is possible to obtain the distribution of energy for that specific amino acid substitution, $m_{i}=E\left(p_{k}∕m_{i}\right)$. This energy distribution accounts for the different energy value a specific substitution may have depending on the external conditions (pH, temperature, pressure, etc.). It also, accounts for how the substitution influences the protein structure in its surroundings.

Data Testing: After completing the learning stage, where the amino acid substitution CDFs are obtained, a testing stage is carried out. This means that every change of energy in the mutant protein $\;E_{cdf,MC,k}$ in the testing bag is predicted with a Monte Carlo algorithm, where the rejection or acceptance of a simulation is based on the previously computed CDF. The energy distribution computed with the Monte Carlo method is compared with the real experimentally measured energy and a residual $\Omega_{k}$ with the weight *k*_Ω_ computed, to fit the following expression:

(1) $$E_{cdf,real,k}=E_{cdf,MC,k}+k_{\Omega }\cdot \Omega_{k,cdf}$$

Holdout selection: Once CDFs of energy change for the mutant protein are computed in the training dataset $T_{k}$, the Holdout accuracy is computed and the best Holdout predictors are chosen to predict, afterwards CDFs of energy changes for the entire protein dataset. To compute the Holdout accuracy, we define the energy change median sign as follows:

(2) $$S_{k,pred}=\frac{median\;\left(E_{cdf,pred,k}\right)}{\left|median\;\left(E_{cdf,pred,k}\right)\right|}\;S_{k,pred}\in \left\{-1,+1\right\}$$

In addition, we compute the sign of the real energy in the training dataset:

(3) $$S_{k,obs}\in \left\{-1,+1\right\}$$

The accuracy is defined as the percentage of proteins, whose predicted energy change sign coincides with the observed energy change sign, as follows:

(4) $$Acc_{HD,i}=\frac{\sum_{k=1}^{n}\{\begin{array}{c} 1\;\;S_{k,pred}=S_{k,obs}\\ 0\;S_{k,pred}\neq S_{k,obs} \end{array}}{n}$$

The Holdouts that fulfill the following condition: $Acc_{HD,i}>0.99\cdot Acc_{HD,min\;}$ are selected to compute the cumulative distribution function of energy changes for the entire protein dataset.

Computing the distribution of protein energy changes: After selecting the best Holdouts that fulfill our threshold condition, the residual distribution, $\Omega_{k,cdf}$ and the weight, $k_{\Omega }$ are averaged throughout the entire holdouts. In this sense, the energy change is predicted with a Monte Carlo algorithm, where the rejection or acceptance of a simulation is based on the holdout learned CDFs and adjusted with the weigh and residual according to the expression:

(5) $$E_{cdf,pred,k}=E_{cdf,MC,k}+k_{\Omega }\cdot \Omega_{k,cdf}$$

This part of the algorithm is general and, prediction of CDFs of energy changes for both Single and Multiple Point Mutations follows the same procedures and equations.

### 2.4. The Neural Network Based Predictor

**Figure 2:**
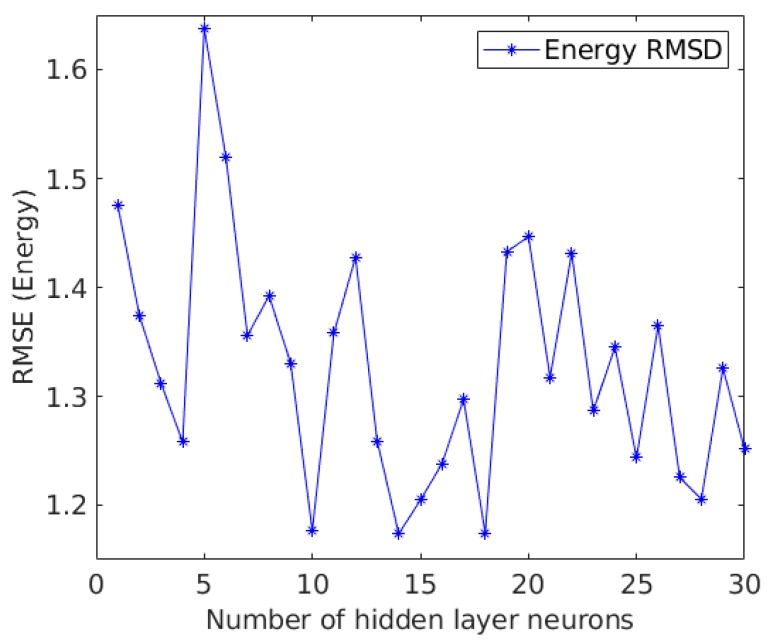
Neural Network Architecture tuning. This analysis was performed with a reduced dataset in order to evaluate the NN performance optimal point.

Artificial neural networks are computing systems composed of simple processors whose layered, interconnected architecture resembles the structure of neurons in the brain. A neural network is capable of learning from data, so it can be trained to recognize patterns, classify data and or perform regressions [46]. A neural network divides the input data into layers of abstraction, and it could be trained over many input datasets to perform predictions. A neural network performance depends on connectivity of individual neurons, their weights and the strengths of these connections. All these parameters are automatically adjusted and updated at every step during the training/learning process. This is carried out until the neural network performs its task (classification, regression, pattern recognition) with a high degree of accuracy for the training/learning dataset [47]. Due to that, neural networks are especially well suited to solve classification and regression problems. The neural network presented in this paper, combines one input layer where 100 parameters corresponding to the CDF, the mutation type and the protein code are provided, one hidden layer with 10 neurons and an output layer with one neuron, which provides the free energy change prediction. The architecture of the Neural Network was found to be optimum when the hidden layer consists of 10 nodes after the analysis (see Figure 2). This tuning was performed with a 20% of the data in order to sample the neural network hidden layer architecture and select the best configuration possible.

The layers are interconnected via nodes with each layer using the output of the previous one as its input. The neural network performs supervised learning, since it is trained in order to produce the desired targets (observed energy) according to a set of inputs (Protein ID, mutation type and CDF of the change of energy). In this sense, the algorithm can perform a classification of the proteins and their mutations while, carrying out a regression by modelling the response between the CDF of the energy change and position and the observed energy in the ProTherm database.

The neural network is trained and validated 100 times by splitting randomly the data set in a way such that 70% of the mutated proteins and their CDFs are used for learning, 15% are used for testing and 15% for blind validation. This Neural Network utilizes the Levenberg-Marquardt algorithm as a training method [48] and RELU activation function.

## 3. Results

**Figure 3:**
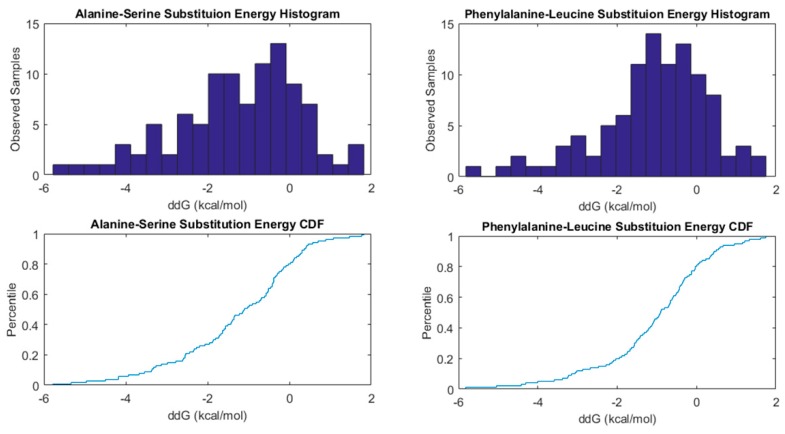
Alanine-Serine and Phenylalanine-Leucine amino-acid substitutions change cumulative distributions of energy changes and frequency histograms. These energy changes are observed in different protein sequences at different positions.

In the ProTherm database, mutations in protein sequence are related to the protein stability change (ΔΔG) Therefore, every single mutation in the database is taken alongside the energy change it causes, in order to construct the associated cumulative distribution of energy changes. Figure 3 shows the cumulative distribution of energy changes for the amino acid substitution A–S (where Alanine is substituted with Serine) and F–L (where Phenylalanine is substituted with Leucine). As it can be observed the problem is highly uncertain, since a wide range of energy changes are observed with a high frequency. Therefore, the prediction of energy changes in protein mutants should always be accompanied by a proper uncertainty analysis and model parametrization to ensure that the models perform according to the highest accuracy standards.

The Holdout Random Sampler was designed in this paper as a consensus classifier. The utilization of consensus classifiers generally leads to significant improvement of prediction performance, and, at the same time, represents a good tool for predicting effects of protein mutations. In this sense, it confirms that consensus prediction is an accurate and robust alternative to classical and individual machine learning tools [49].

Therefore, the Holdout Random Sampler employs a consensus system connecting different decision boundaries. In this sense, the Holdout Random Sampler, removes and does not consider protein mutations with low statistics, which leads to inaccuracies in the evaluation of (ΔΔG) in the CDF. In addition, the Holdout Random Sampler computes the sign of the median (ΔΔG) in the CDF in order to decide whether the specific mutation will lead to protein stability or instability.

The accuracy of each Holdout is computed as reported in the previous section, utilizing the expression:

(6) $$Acc_{HD,i}=\frac{\sum_{k=1}^{n}\{\begin{array}{c} 1\;\;S_{k,pred}=S_{k,obs}\\ 0\;S_{k,pred}\neq S_{k,obs} \end{array}}{n}$$

**Figure 4:**
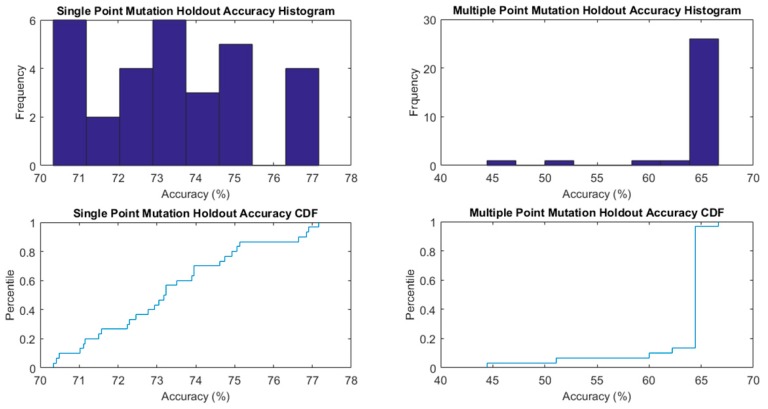
Single Point and Multiple Point Mutation Holdout Random Sampler Accuracy on Testing subset cumulative distributions and frequency histograms.

The results are combined in Figure 4, where both the CDFs of accuracies and distribution histograms are presented.

Once the accuracy of each Holdout is calculated, those which satisfy the condition:

$Acc_{HD,i}>0.99\cdot Acc_{HD,min\;}$ are selected to compute the distribution of energy changes for the entire protein dataset.

This distribution of energy changes is obtained from $E_{cdf,pred,k}=E_{cdf,MC,k}+k_{\Omega }\cdot \Omega_{k,cdf}$, where the parameter $k_{\Omega }$ and the distribution $\Omega_{k,cdf}$ were learned in the Holdouts and predicted by the selected ones. The distribution of energy change $E_{cdf,MC,k}$ is predicted by the best Holdout through a Monte Carlo simulation, in which the acceptance or rejection of an energy change is determined by the landscape of possible energy changes that a mutation causes regardless of the residue, its position or a protein. This energetic landscape for mutations was previously found in the Holdout learning dataset and predicted by the selected best Holdouts.

**Figure 5:**
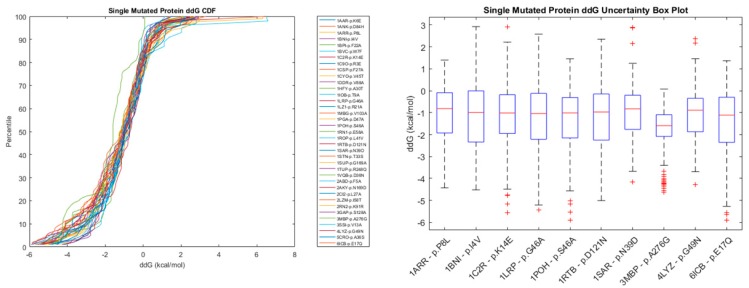
Cumulative distribution function of energy changes and the uncertainty plot for selected proteins with a single point mutation.

**Figure 6:**
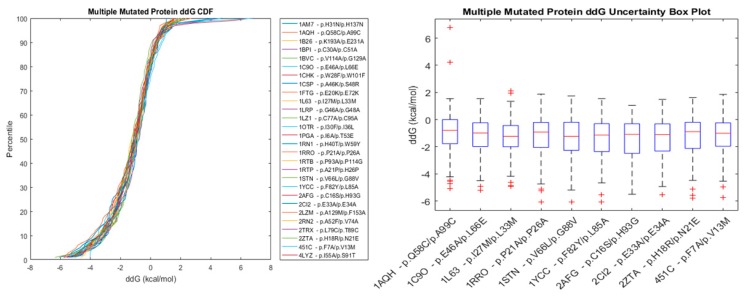
Cumulative distribution function of energy changes and the uncertainty plot for selected proteins with multiple point mutations.

Figure 5 and Figure 6 show the cumulative distribution function of energy changes for selected sets of proteins with a Single Mutation and with Multiple Mutations. It can be observed that a given protein mutation can lead to any energy change that is admissible by the CDF, since such energy change may be affected by various factors, such as structural features, amino acid interactions in the neighborhood of the mutation site, temperature, pH, solubility, etc. The boxplots in Figure 5 and Figure 6, help to visualize the high uncertainty nature of this prediction. Supplementary Material 3 show data for a Single Point Mutation Energy Change CDF, and Supplementary Material 4 show similar data for Double Point Mutations.

**Figure 7:**
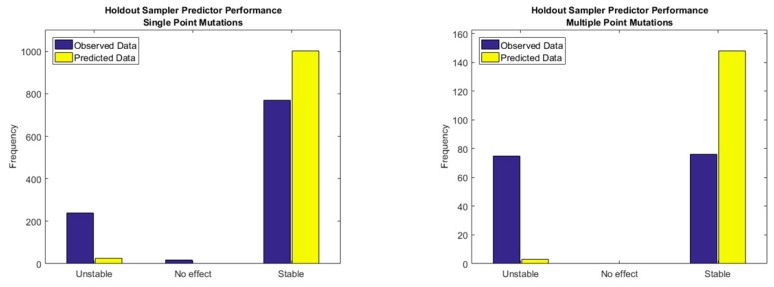
Prediction of Single and Multiple Point Mutations stability via the Holdout Sampler considering the median value of the energy change distribution function.

In a wide range of problems, the median is a promising predictor since it is robust, and it is not highly influenced by outliers. However, the Holdout Random Sampler tends to overpredict stability for protein mutations as shown in Figure 7. This is consistent with the fact that CDF of energy change upon mutation has a much larger region of stability than instability, consequently, during random sampling of the energy landscape of a mutant protein, it is more likely to predict a stable energy change than an unstable one. In that sense, increasing the number of training cases of unstable mutations would contribute to the improvement of the quality of predictions with only the Holdout Sampler, otherwise a posterior regression model, as the one implemented in this research is required.

However, the Holdout Random Sampler was designed and should be understood as a simple methodology to sample the uncertainty space of the energy changes upon mutation for a specific protein, but not to accurately predict the energy change. Therefore, it is a very simple, but powerful tool to utilize the outcome of it to parametrize a Neural Network in order to dramatically reduce its complexity.

Neural Networks experience a highly varying performance which depends on the initial random conditions, when relatively small datasets are utilized. This poor performance is specially found on training datasets, which suggests that their hyper-parameter tuning processes normally underfits, rather than overfits their performance, in contrast with other methods such as Random Forest or Support Vector Machines. Consequently, semi-supervised methods or “a priori” parametrization of the model seems to be the best fit when approaching this problem, which agrees with Dehghanpoor et al. [28].

**Figure 8:**
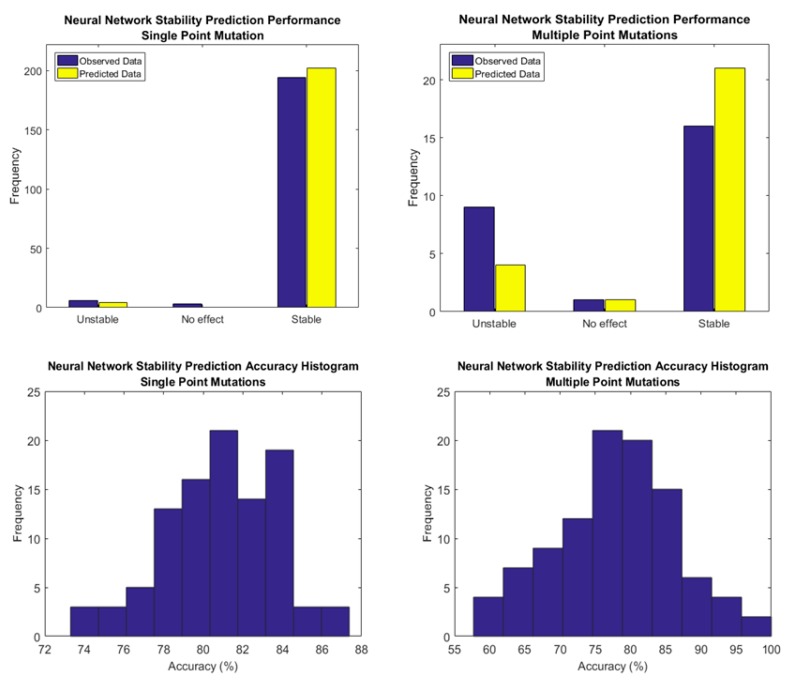
Performance summary of the Neural Network as a stability predictor for Single and Multiple Point Mutations alongside the obtained Accuracy Histogram averaged over 100 simulations.

**Figure 9:**
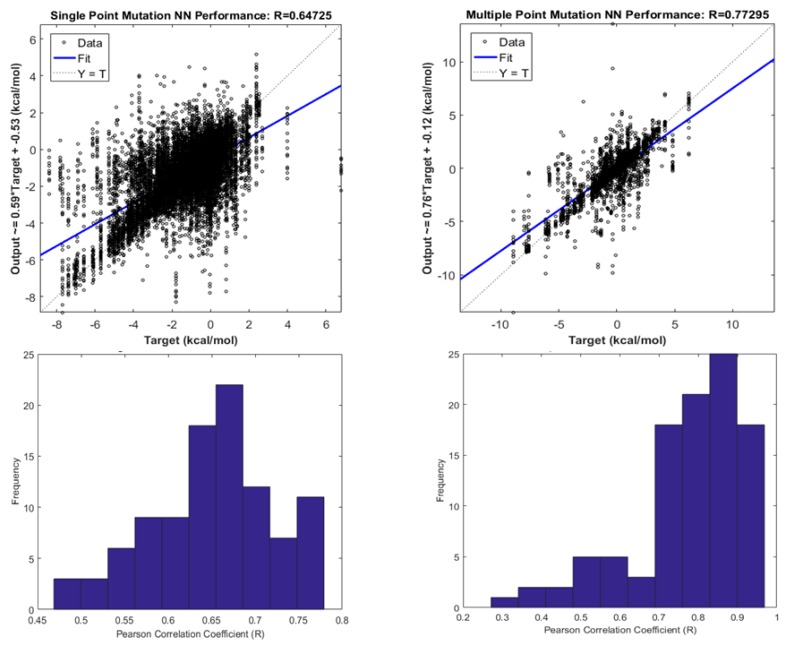
Average Neural Network Performance over randomly selected blind validation subsets and the corresponding Pearson Correlation Coefficient for frequency histograms for both Single Point and Multiple Point Mutations.

**Figure 10:**
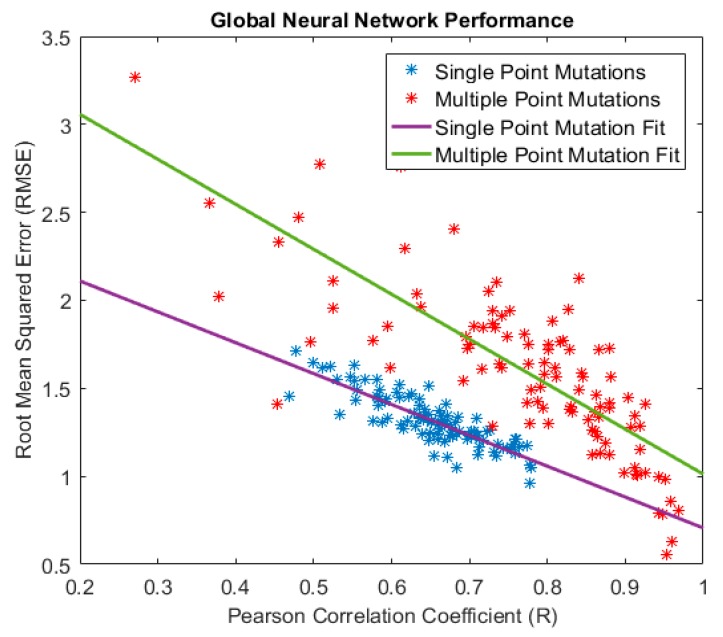
Relationship between RMSE and Pearson Correlation Coefficient for Single and Multiple Point Mutations.

Figure 8, Figure 9 and Figure 10, show the performance of a simple Neural Network composed of 10 nodes utilizing the Levenberg-Marquardt algorithm as the training function. In Figure 8, it is possible to observe how the Neural Network alleviates the tendency to over predict the stability in contrast with the Holdout Random Sampler, giving the average accuracy in the sign prediction 81% for Single Point Mutations and 78% for Multiple Point Mutations. Additional data for Single Point Mutations is shown in Supplementary Material 5, and for Double Point Mutations in Supplementary Material 6.

Figure 9 shows the averages for the training, testing and validation datasets that were randomly split 100 times in a way such that 70% of the mutant proteins and their CDFs were used for learning, 15% were used for testing and 15% were used for blind validation. The Neural Network classifies the protein type, mutation type and performs a regression with the cumulative distribution function of energy change to fit the target values of energy changes.

The assessment of the accuracy of predicting the values of energy changes of mutations in the protein sequence is carried out by analyzing the Pearson Correlation Coefficients and the Root Mean Square Error (RMSE) shown in Figure 10. This figure shows that the Pearson Correlation Coefficient increases linearly as the RMSE decreases, which is expected result. Nevertheless, for the same RMSE value, the Pearson Correlation Coefficient is much higher for the Multiple Point mutations and the slope of the regression line is smaller (has a larger absolute value).

The results presented in Figure 9 and Figure 10 show that our algorithm outperforms other approaches reported [35,50] in the literature and summarized in Table 1.

Our Pearson Correlation Coefficient 0.77 ranks top, followed by ProMaya, Mcsm and ELASPIC servers which have achieved correlation coefficients of 0.74, 0.76, and 0.77 respectively. Supplementary Material 7 shows the raw results from the neural network, which predicts the value of free energy changes upon mutations. Despite having different cross-validation approaches or pre-processing schemes, the results could be reasonably compared as supported by the hypothesis of Biological Invariance reported by Alvarez et al. [61], that is; the analysis of the genomics, metabolomics and proteomics data should be independent of the sampling methodology and the classifier utilized for their inference. Only the differences in the size of the datasets might affect the comparison, since each set has its inherent noise and peculiarities. In addition, the utilization of binary classification data to exclude neutral models (ΔΔG=0±0.5 kcal/mol), might affect the ultimate performance of this prediction scheme. Another major point that is worth mentioning with respect to our prediction methodology is that no direct energetic calculations were performed, the methodology was solely based on generalizing the landscape of energetic changes for each amino acid substitution regardless the residue position and the protein. Later, this generalization was applied to specific proteins in order to obtain the set of admissible values of energy changes to parametrize a neural network

## 4. Conclusions

In this work, we present a pre-parametrized machine learning-based methodology to infer the effects of single and multiple point mutations on the stability of a protein. More specifically, our approach can predict the change of free energy of unfolding upon mutation (ΔΔG) by using the Holdout Random Sampler to compute the distribution of the change of free energy of unfolding upon mutation of a protein and a Neural Network composed of 10 nodes to predict this effect. This distribution consists of a set of admissible values of free energy changes of a protein and it is an indication of the uncertainty behind this prediction, since the change of energy may be affected by a wide range of factors, from structural to external ones, such as temperature, pH, secondary structure, amino acid interactions within the vicinity of the mutation site, etc.

The Neural Network is trained and tested by randomly splitting the data set. This procedure has been repeated 100 times in order to assess the robustness of the modeling. Our average Pearson Correlation coefficient is 0.6630 in the case of single point mutations and 0.7747 in the case of multiple point mutations, which proves that our method predicts the effects of mutations with high accuracy and a low root mean square error (RMSE), outperforming other algorithms currently available in the literature.

## Figures and Tables

**Table 1:** Overall Performance of Holdout-NN sampler in comparison with the performance of other commonly used methods.

Method	Highest Reported Pearson Coefficient (R)	Reference
Holdout-NN Method	0.77	
Regression with RF	0.66	Li et al. [36]
MUpro	0.48	Cheng et al. [10]
I-Mutant 2.0	0.54	Capriotti et al. [51]
LSE	0.16	Jia et al. [35]
FoldX	0.50	Schymkowitz et al. [52]
EGAD	0.60	Pokala et al. [53]
PROTS	0.40	Li et al. [54]
PopMuSiC-2.0	0.62	Dehouck et al. [55]
Prethemut	0.72	Farhoodi et al. [56]
ProMaya	0.74	Wainreb et al. [57]
ELASPIC	0.77	Witvliet et al. [58]
SDM2	0.52	Pandurangan et al. [31]
ENCoM	0.44	Frappier et al. [59]
DynaMut	0.67	Rodrigues et al. [50]
mCSM	0.76	Pires et al. [60]

## Supplementary Materials

The following are available online at https://www.mdpi.com/2218-273X/10/1/67/s1. Supplementary Material 1: Single Point Mutation Dataset, Supplementary Material 2: Double Point Mutation Dataset, Supplementary Material 3: Single Point Mutation Energy Change CDF, Supplementary Material 4: Double Point Mutation Energy Change CDF, Supplementary Material 5: Single Point Mutation NN Results, Supplementary Material 6: Double Point Mutation NN Results. Supplementary Material 5 and Supplementary Material 6 are the raw results from the neural network, which predicts the value of free energy changes upon mutations.

## References

[1] Daggett V., Fersht A.R. (2003). Is there a unifying mechanism for protein folding?. Trends Biochem. Sci..

[2] Casadio R., Compiani M., Fariselli P., Vivarelli F. (1995). Predicting free energy contributions to the conformational stability of folded proteins from the residue sequence with radial basis function networks. Proc. Int. Conf. Intell. Syst. Mol. Biol..

[3] Kumar M., Bava K., Gromiha M., Prabakaran P., Kitajima K., Uedaira H., Sarai A. (2005). ProTherm and Pronit: Thermodynamic databases for proteins and protein-nucleic acid interactions. Nucleic. Acids Res..

[4] Risch N.J. (2000). Searching for genetic determinants in the new millennium. Nature.

[5] Ng P.C., Henikoff S. (2006). Predicting the effects of amino-acid substitutions on protein function. Annu. Rev. Genom. Hum. Genet..

[6] Verma R., Schwaneberg U., Roccatano D. (2012). Computer-aided Protein Directed Evolution: A review of web servers, databases and other computational tools for protein engineering. Comput. Struct. Biotech. J..

[7] Boucher J.I., Bolon D.N.A., Tawfik D.S. (2016). Quantifying and understanding the fitness effects of protein mutations: Laboratory versus nature. Protein Sci..

[8] Gnad F., Baucom A., Mukhyala K., Manning G., Zhang Z. (2013). Assessment of computational methods for predicting the effects of missense mutations in human cancers. BMC Genom..

[9] Capriotti E., Fariselli P., Casadio R. (2004). A neural network-based method for predicting protein stability changes upon single point mutations. Bioinformatics.

[10] Cheng J., Randall A., Baldi P. (2006). Prediction of Protein Stability Changes for Single-Site Mutations Using Support Vector Machines. Proteins.

[11] Guerois R., Nielsen J.E., Serrano L. (2002). Predicting changes in the stability of proteins and protein complexes: A study of more than 1000 mutations. J. Mol. Biol..

[12] Lee C. (1995). Testing homology modeling on mutant proteins: Predicting structural and thermodynamic effects in the ala98-val mutants of t4 lysozyme. Fold. Des..

[13] Zhou H., Zhou Y. (2002). Distance-scaled, finite ideal-gas reference state improves structure-derived potentials of mean force for structure selection and stability prediction. Protein Sci..

[14] Sippl M.J. (1995). Knowledge based potentials for proteins. Curr. Opin. Stuct. Biol..

[15] Prevost M., Wodak S., Tidor B., Karplus M. (1991). Contribution of the hydrophobic effect to protein stability: Analysis based on simulations of the Ile-96-Ala mutation in barnase. Proc. Natl. Acad. Sci. USA.

[16] Topham C.M., Srinivasan N., Blunell T.L. (1997). Prediction of the stability of protein mutants based on structural environment-dependent amino-acids substitution and propensity tables. Protein Eng..

[17] Zhou H., Zhou Y. (2004). Quantifying the effect of burial of amino-acid residues on protein stability. Proteins.

[18] Gillis D., Rooman M. (1997). Predicting protein stability changes upon mutation using database-derived potentials: Solvent accesibility determines the importance of local versus non-local interactions along the sequence. J. Mol. Biol..

[19] Carter C.W., LeFebvre B.C., Cammer S.A., Torpsha A., Edgell M.H. (2001). Four body potentials reveal protein specific correlations to stability changes caused by hydrophobic core mutations. J. Mol. Biol..

[20] Takano K., Ota M., Ogasahara K., Yamagata Y., Nishikawa K., Yutani K. (1999). Experimental verification of the stability profile of mutant protein [spmp) data using mutant human lysozymes. Protein Eng..

[21] Domingues H., Peters J., Schneider K.H., Apeler H., Sebald W., Oschkinat H., Serrano L. (2000). Improving the refolding yield of interleukin-4 through the optimization of local interactions. J. Biotechnol..

[22] Funahashi J., Takano K., Yutani K. (2001). Are the parameters of various stabilization factors estimated from mutant human lysozymes compatible with other proteins?. Protein Eng..

[23] Radestock S., Gohlke H. (2008). Exploiting the Link between Protein Rigidity and Thermostability for Data Driven Protein Engineering. Eng. Life Sci..

[24] Jacobs D., Rader A., Thorpe M., Kuhn L. (2001). Protein Flexibility Predictions Using Graph Theory. Proteins.

[25] Fox N., Jagodzinski F., Li Y., Streinu I. (2011). KINARI-Web: A server for protein rigidity analysis. Nucleic Acids Res..

[26] Jagodzinski F., Hardy J., Streinu I. (2012). Using rigidity analysis to probe mutation-induced structural chagnes in proteins. J. Bioinf. Comput. Biol..

[27] Jagodzinski F., Akbal-Delibas B., Haspel N. An evolutionary Conservation & Rigidity Analysis Machine Learning Approach for Detecting Critical Protein Residues. Proceedings of the ACM International Conference on Bioinformatics, Computational Biology and Biomedical Informatics (ACM-BCB).

[28] Dehghanpoor R., Ricks E., Hursh K., Gunderson S., Farhoodi R., Haspel N., Hutchinson B., Jagodzinski F. (2018). Predicting the Effect of Single and Multiple Mutations on Protein Structural Stability. Molecules.

[29] Worth C., Preissner R., Blundell L. (2011). SDM—A server for predicting effects of mutations on protein stability and malfunction. Nucleic Acids Res..

[30] Brender J.R., Zhang Y. (2015). Predicting the effect of mutations on protein-protein binding interactions through structure-based interface profiles. PLoS Comput. Biol..

[31] Pandurangan A.P., Ochoa-Montaño B., Ascher D.B., Blundell T.L. (2017). SDM: A server for predicting effects of mutations on protein stability. Nucleic Acids Res..

[32] Wei L., Xing P., Shi G., Ji Z., Zou Q. (2019). Fast prediction of protein methylation sites using a squence-based feature selection technique. IEEE/ACM Trans. Comput. Biol. Bioinform..

[33] Wei L., Xing P., Tang J., Zou Q. (2017). PhosPred-RF: A novel Sequence Based Predictor for Phosphorylation Sites using Sequential Information Only. IEEE Trans. Nanobiosci..

[34] Wan S., Duan Y., Zou Q. (2017). HPSLPred: An Ensemble Multi-Label Classifier for Human Protein Subcellular Location Prediction with Imbalanced Source. Proteomics.

[35] Jia L., Yarlagadda R., Reed C.C. (2015). Structure Based Thermostability Prediction Models for Protein Single Point Mutations with Machine Learning Tools. PLoS ONE.

[36] Li Y., Fang J. (2012). PROTS-RF: A robust model for predicting mutation-induced protein stability changes. PLoS ONE.

[37] Wolpert D.H. (1992). Stacked generalization. Neural Netw..

[38] Breiman L. (1996). Stacked regressions. Mach. Learn..

[39] LeBlanc M., Tibshirani R. (1996). Combining estimates in regression and classification. J. Am. Stat. Assoc..

[40] Fernández-Martínez J.L., Fernández-Muñiz Z., Breysse D. (2018). The uncertainty analysis in linear and nonlinear regression revisited: Application to concrete strength estimation. Inverse Probl. Sci. Eng..

[41] Fernández-Muñiz Z., Hassan K., Fernández-Martínez J.L. (2019). Data kit inversion and uncertainty analysis. J. Appl. Geophys..

[42] Fernández-Martínez J.L., Cernea A., deAndrés-Galiana E.J., Fernández-Ovies F.J., Fernández-Muñiz Z., Alvarez-Machancoses O., Saligan L., Sonis S.T. Sampling Defective Pathways in Phenotype Prediction Problems via the Holdout Sampler. Bioinformatics and Biomedical Engineering. Proceedings of the International Conference on Bioinformatics and Biomedical Engineering IWBBIO 2018.

[43] Abdulla B.K., Gromiha M.M., Uedaira H., Kitajima K., Sarai A. (2004). ProTherm, version 4.0: Thermodynamic database for proteins and mutants. Nucleic Acids Res..

[44] Berman H.M., Henrick K., Nakamura H. (2003). Announcing the worldwide Protein Data Bank. Nat. Struct. Biol..

[45] Efron B., Tibshirani R. (1993). An Introduction to the Bootstrap.

[46] Jain A.K., Mao J., Mohiuddin K.M. (1996). Artificial Neural Networks: A tutorial. Computer.

[47] Wasserman P.D. (1993). Advanced Methods in Neural Computing.

[48] Moré J.J. (1978). The Levenberg-Marquardt algorithm: Implementation and theory. Numer. Anal..

[49] Bendl J., Stourac J., Salanda O., Pavelka A., Wieben E.D., Zendulka J., Brezovsky J., Damborsky J. (2014). Predict SNP: Robust and Accurate Consensus Classifier for Prediction of Disease-Related Mutations. PLoS Comput. Biol..

[50] Rodrigues C.H.M., Pires D.E.V., Ascher D.B. (2018). DynaMut: Predicting the impact of mutations on protein conformation, flexibility and stability. Nucleic Acids Res..

[51] Capriotti E., Fariselli P., Casadio R. (2005). I-Mutant2.0: Predicting stability changes upon mutation from the protein sequence or structure. Nucleic Acids Res..

[52] Schymkowitz J., Borg J., Stricher F., Nys R., Rousseau F., Serrano L. (2005). The FoldX web server: An online force field. Nucleic Acids Res..

[53] Pokala N., Handel T.M. (2005). Energy functions for protein design: Adjustment with protein–protein complex affinities, models for the unfolded state, and negative design of solubility and specificity. J. Mol. Biol..

[54] Li Y., Zhang J., Tai D., Middaugh C.R., Zhang Y., Fang J. (2012). Prots: A fragment based protein thermo-stability potential. Proteins Struct. Funct. Bioinform..

[55] Dehouck Y., Grosfils A., Folch B., Gilis D., Bogaerts P., Rooman M. (2009). Fast and accurate predictions of protein stability changes upon mutations using statistical potentials and neural networks: PoPMuSiC-2.0. Bioinformatics.

[56] Farhoodi R., Shelbourne M., Hsieh R., Haspel N., Hutchinson B., Jagodzinski F. (2017). ACM. Predicting the Effect of Point Mutations on Protein Structural Stability. Comput. Biology Health Inform..

[57] Wainreb G., Wolf L., Ashkenazy H., Dehouck Y., Ben-Tal N. (2011). Protein stability: A single recorded mutation aids in predicting the effects of other mutations in the same amino acid site. Bioinformatics.

[58] Witvliet D.K., Strokach A., Giraldo-Forero A.F., Teyra J., Colak R., Kim P.M. (2016). ELASPIC web-server: Proteome-wide structure-based prediction of mutation effects on protein stability and binding affinity. Bioinformatics.

[59] Frappier V., Chartier M., Najmanovich R.J. (2015). ENCoM server: Exploring protein conformational space and the effect of mutations on protein function and stability. Nucleic Acids Res..

[60] Pires D.E., Ascher D.B., Blundell T.L. (2013). mCSM: Predicting the effects of mutations in proteins using graph-based signatures. Bioinformatics.

[61] Alvarez O., Fernández-Martínez J.L. (2019). The importance of Biological Invariance in Drug Design. Biomed. J. Sci. Tech. Res..

